# Utility Scores for Risk-Reducing Mastectomy and Risk-Reducing Salpingo-Oophorectomy: Mapping to EQ-5D

**DOI:** 10.3390/cancers16071358

**Published:** 2024-03-30

**Authors:** Samuel G. Oxley, Xia Wei, Michail Sideris, Oleg Blyuss, Ashwin Kalra, Jacqueline J. Y. Sia, Subhasheenee Ganesan, Caitlin T. Fierheller, Li Sun, Zia Sadique, Haomiao Jin, Ranjit Manchanda, Rosa Legood

**Affiliations:** 1 Wolfson Institute of Population Health, Queen Mary University of London, London EC1M 6BQ, UK; s.oxley@qmul.ac.uk (S.G.O.); xia.wei@lshtm.ac.uk (X.W.); m.sideris@qmul.ac.uk (M.S.); oleg.blyuss@qmul.ac.uk (O.B.); a.kalra@qmul.ac.uk (A.K.); j.sia@qmul.ac.uk (J.J.Y.S.); s.ganesan@qmul.ac.uk (S.G.); c.fierheller@qmul.ac.uk (C.T.F.); 2 Department of Gynaecological Oncology, Barts Health NHS Trust, Royal London Hospital, London E1 1BB, UK; 3 Department of Health Services Research and Policy, London School of Hygiene & Tropical Medicine, London WC1H 9SH, UK; li.sun1@lshtm.ac.uk (L.S.); zia.sadique@lshtm.ac.uk (Z.S.); 4 Department of Pediatrics and Pediatric Infectious Diseases, Institute of Child´s Health, Sechenov First Moscow State Medical University, Sechenov University, Moscow 119991, Russia; 5 School of Health Sciences, Faculty of Health and Medical Sciences, University of Surrey, Guildford GU2 7YH, UK; h.jin@surrey.ac.uk; 6 MRC Clinical Trials Unit at UCL, Institute of Clinical Trials & Methodology, Faculty of Population Health Sciences, University College London, London WC1V 6LJ, UK

**Keywords:** risk-reducing mastectomy, risk-reducing salpingo-oophorectomy, utility scores, mapping

## Abstract

**Simple Summary:**

Surgery to prevent breast and ovarian cancer is very effective, but there is limited research into the quality of life afterwards, particularly with reference to quality-of-life scores known as utility scores. These are important for patients at increased cancer risk who are considering surgery and are essential for health-economic evaluations. This article used data from a systematic review of different quality-of-life questionnaires given to patients following these surgeries. We converted these data into utility scores, as recommended by the national guidelines. This shows that surgery to prevent breast cancer is associated with a long-term utility score of 0.92 and surgery to prevent ovarian cancer has a score of 0.97, indicating a mild–moderate impact. These are the first utility scores sourced from patients who have undergone these procedures, and they are important to doctors counselling patients about cancer prevention options and to researchers. Higher-quality studies are still needed, using the recommended quality-of-life questionnaire (EQ-5D).

**Abstract:**

Background: Risk-reducing mastectomy (RRM) and risk-reducing salpingo-oophorectomy (RRSO) are the most effective breast and ovarian cancer preventive interventions. EQ-5D is the recommended tool to assess the quality of life and determine health-related utility scores (HRUSs), yet there are no published EQ-5D HRUSs after these procedures. These are essential for clinicians counselling patients and for health-economic evaluations. Methods: We used aggregate data from our published systematic review and converted SF-36/SF-12 summary scores to EQ-5D HRUSs using a published mapping algorithm. Study control arm or age-matched country-specific reference values provided comparison. Random-effects meta-analysis provided adjusted disutilities and utility scores. Subgroup analyses included long-term vs. short-term follow-up. Results: Four studies (209 patients) reported RRM outcomes using SF-36, and five studies (742 patients) reported RRSO outcomes using SF-12/SF-36. RRM is associated with a long-term (>2 years) disutility of −0.08 (95% CI −0.11, −0.04) (I^2^ 31.4%) and a utility of 0.92 (95% CI 0.88, 0.95) (I^2^ 31.4%). RRSO is associated with a long-term (>1 year) disutility of −0.03 (95% CI −0.05, 0.00) (I^2^ 17.2%) and a utility of 0.97 (95% CI 0.94, 0.99) (I^2^ 34.0%). Conclusions: We present the first HRUSs sourced from patients following RRM and RRSO. There is a need for high-quality prospective studies to characterise quality of life at different timepoints.

## 1. Introduction

The number of women identified to be at a high lifetime risk of breast cancer (BC) and ovarian cancer (OC) is increasing due to the expansion in genetic testing provision after cancer diagnosis [1], with new programmes offering BRCA population testing in Jewish individuals [2,3] and the lowering of thresholds of genetic testing [4] making it far more accessible than earlier. Most of the cancer susceptibility gene carriers in the population are yet to be detected [5]. Women may be at high risk due to a family history of cancer or a pathogenic or likely pathogenic variant in a cancer-susceptibility gene (such as *BRCA1*, *BRCA2*, or *PALB2*). Risk-reducing surgery is the most effective BC and OC preventive strategy and is recommended for high-risk women after careful counselling [6,7]. Risk-reducing mastectomy (RRM) results in an 89–96% [8,9,10] BC risk reduction and may be combined with immediate or delayed reconstruction. Risk-reducing salpingo-oophorectomy (RRSO) provides a 96% OC risk reduction [11] and a decreased BC risk in *BRCA2* carriers [12]. RRSO results in a definitive end to natural fertility and a surgical menopause for pre-menopausal women, which can be treated with hormone-replacement therapy (HRT) to provide symptomatic relief and prevent long-term health effects [13,14]. RRM and RRSO both result in a short-term impact on health-related quality-of-life (QoL) during recovery and may affect QoL outcomes in the medium to long term.

It is important for clinicians counselling patients at increased cancer risk to understand the level of any QoL impact from risk-reducing surgery. It is also essential for health-economic evaluations, which determine the most equitable and appropriate use of finite resources within a health service. QoL can be described on a scale from 1 (perfect health) to 0 (death), known as a health-related utility score (HRUS). Utility scores provide information on the average QoL for a given health condition (such as following risk-reducing surgery). These can either be expressed as a disutility (e.g., −0.1) that is added to an individual’s baseline quality-of-life (additive method), or a utility score (e.g., 0.9) that is multiplied by their baseline level (multiplicative method). The HRUS can be multiplied by years lived to determine quality-adjusted life-years (QALYs), used in health-economic evaluations to compare the cost per QALY for different treatment strategies [15]. Health-economic evaluations of BC and OC prevention strategies are highly sensitive to the HRUSs of RRM and RRSO, such that small variations in the HRUSs can substantially change the results [16,17,18,19].

The EQ-5D is the guideline-recommended measure to obtain HRUSs in many countries including the UK and USA [15,20], yet there are no EQ-5D HRUSs in the literature for RRM or RRSO. The published HRUSs on RRM and RRSO used by health-economic evaluations of BC/OC prevention derive from two vignette studies of women from the general population and *BRCA* carriers [21,22]. These do not report outcomes from patients who have undergone the procedures, and they are at a potential risk of bias [23] despite their widespread use, as details of the vignette development process are not described. There is, therefore, a need for HRUSs for RRM and RRSO obtained from patients who have undergone these procedures.

When prospective data in patients are not available, mapping from other questionnaires should be considered [24]. Our recent systematic review and meta-analysis reports the QoL outcomes following RRM and RRSO in patients at increased risk of BC/OC [25]. This review found no study that has reported post-operative outcomes using the EQ-5D or any alternative questionnaire for deriving HRUSs such as the SF-6D. Nevertheless, a number of studies reported QoL outcomes using the 36-item short-form survey (SF-36) or the 12-item short-form survey (SF-12). The SF-36 is a generic measure of physical and mental health and has been widely used to compare populations across a range of health states [26]. The eight domains are correlated with two overall measures: the physical component summary (PCS-36) and the mental component summary (MCS-36). The SF-12 contains a subset of 12 questions from the SF-36 and produces the same summary scores (PCS-12 and MCS-12). These SF-12 summary scores have been shown to be very good predictors of the SF-36 summary scores (with means and quartiles differing by 0–1.5 points/100 amongst the general population in the US and nine European countries) [27,28]. Whilst these questionnaires were not designed to generate HRUSs, several mapping algorithms have been published to derive HRUSs from SF-36/SF-12 summary scores [29]. 

This study aims to summarise the best available evidence on EQ-5D values after risk-reducing surgery for BC and OC prevention (RRM and RRSO). Its secondary aims are to obtain different HRUSs for RRM and RRSO at different timepoints, for different population cohorts.

## 2. Materials and Methods

### 2.1. Data Sources

Studies investigating quality of life after RRM or RRSO were identified in our recent systematic review, the details of which have been previously published [25]. In brief, major databases were searched from inception to February 2023. The studies included were those that described women with an increased BC/OC risk due to pathogenic variants in cancer susceptibility genes (typically *BRCA1* or *BRCA2*) or a family history of BC/OC. The intervention was RRM or RRSO. The studies excluded were those that described women with a personal history of BC (for RRM) or OC (for RRSO). The outcomes were QoL outcomes including HRUSs.

The data extracted from each study included the means and mode of variance (standard deviations or 95% confidence interval [95% CI]) of results of validated QoL questionnaires. Where SF-36 dimension scores were provided without summary scores, the physical component summary (PCS) and mental component summary (MCS) scores were calculated [30,31]. To maximise the available data from the included studies, an estimate of the standard deviation of these measures was taken from the country-specific general population [28] when studies lacked this information. Where possible, the data were extracted across different subgroups: (1) long term vs. short term, defining long-term follow-up as ≥1 year for RRSO and ≥2 years for RRM; (2) pre-menopausal vs. post-menopausal RRSO; and (3) pre-menopausal RRSO with HRT vs. no HRT use. 

### 2.2. Mapping Algorithms to EQ-5D

A number of mapping algorithms have been developed to map various QoL instruments to the EQ-5D utility scores [29,32]. For SF-12, we selected the 2-variable mapping algorithm developed by Lawrence et al. [33], which used a large US dataset (the 2000 Medical Expenditure Panel Survey) of 15,662 individuals who completed both the EQ-5D and the SF-12. The authors used linear regression to derive the EQ-5D HRUSs from the PCS-12 and MCS-12 data, with 50% of the data subsequently reserved for validation. This algorithm was chosen given its applicability to the general population using aggregate data (i.e., PCS and MCS), its large sample size, and its predictive ability across a diverse set of subgroups, including its close fit for mild rather than severe health states.

Although several mapping algorithms were considered from SF-36, these were unsuitable for use on aggregate data or contained errors that meant it was not possible to follow the algorithm as published [34], despite contacting the research team for clarifications. Given the equivalence between the SF-36 and SF-12 summary scores [27,28], and the benefits of minimising variance from multiple algorithms, the Lawrence algorithm was used to generate the mean (effect size) and 95% CI of the EQ-5D utility scores from the SF-36 PCS and MCS.

Mapping algorithms from other validated questionnaires with extractable data included in our systematic review and meta-analysis were reviewed and considered [29], including those from the Sexual Activity Questionnaire, the Hospital Anxiety and Depression Scale, the Menopause Rating Scale, the Functional Assessment of Cancer Therapy-Endocrine Subscale, the Impact of Events Scale, the Body Image Scale, and the EORTC Core Quality-of-Life Questionnaire-C30. However, mapping functions to the EQ-5D were unavailable for many, did not demonstrate consistent performance [35], or were validated in inappropriate patient populations for our purposes [29] (e.g., mental health [36], lung cancer [37]). Therefore, only the results from SF-12/SF-36 were considered further. 

### 2.3. Derivation of HRUSs

The PCS-12/36 and MCS-12/36 scores were obtained from each study arm and inputted into the “2-variable model” algorithm [33], given as follows:

$$M_{E}=(0.01411\times PCS)+(0.00967\times MCS)\;–0.3720$$

where *M_E_* is the predicted mean EQ-5D score; 0.01411 is the PCS coefficient; 0.00967 is the MCS coefficient; and −0.3720 is the intercept. The 95% confidence intervals for the HRUSs were calculated using the standard deviation for the PCS and MCS and the sample size for each study arm (see original publication for formulae [33]).

### 2.4. Comparison Groups

Where studies provided data from a control group, these were used for comparison. Where there was no control group, the reference HRUSs were obtained from the literature for the female general population within the same age range for relevant countries: Sweden [38], Germany [39], the Netherlands [39], South Korea [39], Canada [40], and the US [39].

### 2.5. Data Analysis

A meta-analysis was conducted to obtain the aggregated unadjusted HRUSs for RRM and RRSO. A random-effects meta-analysis was used to account for the pre-anticipated heterogeneity across the different cohorts. Although heterogeneity was quantified using I^2^, this did not alter the initial assumption that the included cohorts may be significantly heterogenous due to their different countries of origin and other factors. The random-effects meta-analysis was used to pool the HRUSs for RRM and RRSO, adjusted for study control arm/age- and country-specific population reference. Where population reference values were used, the sample size was given as that for the surgical arm, in order not to bias the weighting. The resultant variance was calculated based on the variance/sample size of the original cohorts. Two methodologies are reported:

1. Additive approach, where the disutility from RRS is provided as a utility decrement (HRUSs from the control/reference population subtracted from those from the surgical arm).
2. Multiplicative approach, where the utility score from RRS is provided as a co-efficient (HRUSs from the population undergoing surgery divided by those from the control/reference population).

Further subgroup (sensitivity) analyses were undertaken to derive EQ-5D utilities for (1) the timepoint after surgery, including the first 2 years after RRM vs. after, and the first year after RRSO vs. after; (2) pre-menopausal vs. post-menopausal women after RRSO; (3) pre-menopausal RRSO with HRT vs. no HRT use. The data were extracted in tables using Excel version 2402 (Microsoft, Redmond, WA, USA), and Stata 16 (Statacorp, College Station, TX, USA) was used for statistical analysis.

## 3. Results

### 3.1. Included Studies

No studies reported EQ-5D outcomes in patients following RRM or RRSO. Four observational studies [41,42,43,44] reported on quality of life after RRM using SF-36. Summary scores (PCS-36 and MCS-36) were available in one study [43], whereas the other three studies reported dimension scores only, and hence, the summary scores were calculated [41,42,44]. The standard deviations for PCS-36/MCS-36 were estimated in all four studies. For RRSO, five studies reported data using SF-36 [45,46,47,48,49], of which two studies reported summary scores [45,46] and the others reported dimensions, and hence, the summary scores were calculated. The standard deviations were provided in one of these studies [45] and were estimated in the others. In addition, one study reported the SF-12 summary scores [50], and the standard deviations for PCS-12/MCS-12 were estimated.

### 3.2. Study Arms

For RRM, all four studies were cohorts with no control arm. One study (48 patients) provided outcomes under two years’ follow-up only [43], one study (92 patients) reported outcomes under 2 years’ and over 2 years’ follow-up [41], and two studies (22 patients, 47 patients) reported outcomes over 2 years’ follow-up only [42,44].

For RRSO, four studies provided outcomes from a control arm, and two were cohorts with no control arm [46,47,48,50]. All six studies provided the outcomes of cohorts at over a 1-year follow-up. Two studies (38 patients, 528 patients) additionally provided outcomes under a 1-year follow-up [46,47]. Two studies (30 patients, 93 patients) provided separate outcomes for pre-menopausal and post-menopausal cohorts, both of which were over a 1-year follow-up [45,51]. It was not possible to extract data on pre-menopausal women separately for HRT use or no HRT use. 

### 3.3. Calculation of HRUSs

The EQ-5D utility scores with 95% confidence intervals were calculated for each study arm based on the PCS and MCS [33]. The population reference HRUSs were obtained along with the standard deviation or standard error, as published. One pilot study on RRSO (12 patients) [49] included biases (very small sample size, control group with different age range), which meant it was not possible to calculate the EQ-5D utility scores without disproportionately affecting the overall results, and it was, therefore, excluded from further analysis. Table 1 summarises the included studies with the calculated utility scores. 

### 3.4. Meta-Analysis of Utility Scores after RRM

The unadjusted utility score over 2 years after RRM (161 patients) was 0.84 (95% CI 0.81, 0.87). The adjusted utility decrement was −0.08 (95% CI −0.11, −0.04), and the adjusted utility score (multiplicative) was 0.92 (95% CI 0.88, 0.95). For follow-up under 2 years (140 patients), the overall unadjusted HRUS was 0.88 (95% CI 0.85, 0.91), with a trend towards a disutility of −0.04 (95% CI −0.07, 0.00) and an adjusted HRUS (multiplicative) of 0.96 (95% CI 0.92, 1.00), which was not significant. Table 2 summarises the meta-analysis results by subgroup. See Supplementary Figure S1a–d for forest plots.

### 3.5. Meta-Analysis of Utility Scores after RRSO

The unadjusted HRUS over 1 year following RRSO (527 patients) was 0.83 (95% CI 0.81, 0.85). The adjusted disutility was −0.03 (95% CI −0.05, 0.00), and the adjusted HRUS (multiplicative) was 0.97 (95% CI 0.94, 0.99). For follow-up under 1 year (566 patients), the unadjusted HRUS was 0.82 (95% CI 0.80, 0.84), the adjusted disutility was −0.04 (95% CI −0.06, −0.01), and the adjusted HRUS was 0.96 (95% CI 0.93, 0.98).

When considering pre-menopausal women (with over 1-year follow-up) (79 patients), the unadjusted HRUS was 0.84 (95% CI 0.79, 0.89), the adjusted disutility was −0.05 (95% CI −0.11, 0.00), and the adjusted HRUS was 0.94 (95% CI 0.89, 0.99). For post-menopausal women (44 patients), the unadjusted HRUS was 0.77 (95% CI 0.70, 0.83), the utility decrement was −0.11 (95% CI −0.19, −0.04), and the adjusted HRUS was 0.87 (95% CI 0.90, 0.95). Table 2 summarises the meta-analysis results by subgroup. 

## 4. Discussion

### 4.1. Summary of Results

This mapping study obtaining EQ-5D utility scores from aggregate published SF-12/SF-36 data finds that RRM is associated with a long-term disutility of −0.08/utility score of 0.92, whilst RRSO is associated with a long-term disutility of −0.03/adjusted utility score of 0.97. RRM is associated with a short-term (under 2 year) trend towards a disutility of −0.04/utility score of 0.96 and RRSO with a short-term (under 1 year) disutility of −0.04/utility score of 0.96. Pre-menopausal RRSO results in an adjusted disutility of −0.05/utility score of 0.94, whereas the available evidence for post-menopausal RRSO suggests an adjusted disutility of −0.11/utility score of 0.87. Some of these analyses involved larger cohorts (≥140 for RRM, ≥527 for RRSO), whilst other subgroup analyses involved much smaller numbers (e.g., 44 for post-menopausal RRSO). This has implications for interpretation, as discussed below.

### 4.2. Strengths and Limitations

This study is the first to report HRUSs sourced from patients who have undergone RRM and RRSO. It follows a previously published high-quality and comprehensive systematic review with a prospectively registered protocol, which followed the PRISMA reporting guidelines [25]. 

There are, however, some limitations, which may affect the interpretation of results. There are small sample sizes for certain cohorts, particularly for RRM and for the RRSO subgroup analysis by pre- vs. post-menopausal status, which limits confidence in these findings. We assume there is heterogeneity between and in some cases within studies, as the cohorts were drawn from different countries with different healthcare systems; to mitigate this, we used random-effects meta-analysis. The cohort studies that included a control arm were not always well matched for age and other characteristics [45], which can introduce bias. In particular, one of the two studies that included post-menopausal women did not report the mean age of the RRSO arm [45], which limits confidence in this subgroup analysis.

Additionally, where a general population reference value was used in lieu of a control arm, this may not be representative of women with potential disutility from a high BC/OC risk status. Thus, comparisons against a healthy general population reference may tend to exaggerate the disutilities from risk-reducing surgery.

Furthermore, there is variation in country-specific general population utility scores in the literature, although a single source was used where possible for consistency. It was not possible to report on the impact of HRT after RRSO, though this is known to significantly affect the QoL [13,14]. The use of mapping from SF-12/36 to EQ-5D is necessarily inferior to the “reference case” methodology of direct administration of the EQ-5D to patients prospectively at baseline and post-operative intervals. We considered the mapping algorithm to be the best available in the literature for aggregate SF-12/36 data, but this, nevertheless, does not account for all the variance between EQ-5D values. Individual patient data were not available, and therefore, summary data were used. Access to individual patient data would enable more accurate mapping.

We did not include any papers reporting relevant outcomes after risk-reducing early salpingectomy, as the full results of large prospective trials have not yet been reported [52]. Nevertheless, this is of significant interest to women at high OC risk, and initial data indicate improved sexual function and menopausal symptoms as well as high satisfaction and acceptability after risk-reducing early salpingectomy [53,54]. This is a potential limitation to this analysis and needs to be addressed in the future.

### 4.3. Interpretation

The results of this study are highly relevant to high-risk patients and their clinicians considering risk-reducing surgery, as well as for health-economic evaluations of BC and OC prevention. The finding of a long-term HRUS following RRSO of 0.97 is similar though slightly higher to that previously reported by Grann and colleagues (0.95) in vignette studies of *BRCA* carriers and controls [22], used as the basis of many health-economic evaluations [17]. The long-term HRUS following RRM of 0.92 is also slightly higher than that previously reported in the same study (0.88). Given that the current study uses prospective data collected from patients undergoing the procedure, these data are complimentary and may be superior to that from a vignette study. 

However, the mild disutility seen is likely not sensitive to the huge variation in QoL outcomes including psychosexual impact reported after RRSO [55,56], particularly for pre-menopausal women who do not take HRT (due to contraindications or other reasons). The SF-12/36/EQ-5D does not directly ask questions on sexual function or menopause-specific QoL and, thus, may underestimate the QoL impact from RRSO on these domains in pre-menopausal women.

The short-term utility score reported after RRM and RRSO (both 0.96) is relatively mild and is higher than the long-term utility score from RRM. This may underestimate the impact of RRM in particular, which is known to require a much longer period of recovery and be associated with a relatively high risk of complications of up to 30% [57]. Health-economic models may use a shorter post-operative timepoint than that reported in this study to better capture this short-term disutility, such as a 1-year cycle length. 

The finding of a lower disutility in the present study is encouraging, in that patients may be reassured that risk-reducing surgery may not lead to as severe a long-term QoL impact. This may encourage some patients to opt for preventive surgery over other less-effective strategies such as breast screening with annual MRI, which may help address the under-utilisation of risk-reducing surgery [58]. However, decision-making in this context is highly complex, and many other factors influence and are associated with preventive behaviours including perceived cancer risk, family history of cancer, personal history of cancer, fertility issues, menopause, and other psychological measures [53,59].

The most direct application of these findings is in health-economic evaluations of BC and OC prevention, which inform clinical guidelines. HRUSs after risk-reducing surgery are a key variable that can significantly impact the result of any health-economic analysis that incorporates surgical prevention (including assessments of non-surgical alternatives) [18,19]. Previous health-economic evaluations of OC and BC prevention may have used more conservative (i.e., larger) disutility values than we find in our analysis. Incorporating a smaller long-term disutility from RRSO may tend to lower the lifetime OC risk threshold at which RRSO is cost effective [18,19]. This is highly relevant for intermediate-risk women and for clinicians and clinical guideline groups in considering the lifetime cancer risk threshold at which to recommend RRSO [4,60]. It is, however, unlikely to influence the results of a cost-effectiveness analysis of the highest-risk (*BRCA1* and *BRCA2*) carriers, as RRM and RRSO is highly cost effective in these patients [61].

Accurate precision around the disutility of RRM and RRSO is essential to understand the short- and long-term impacts on patients. Future prospective studies using the EQ-5D are required to obtain reference values for each procedure. The PROTECTOR trial is recruiting UK women at increased OC risk and administers the EQ-5D-5L at baseline and at post-operative intervals (3 months and thereafter yearly for 3 years) after RRSO and risk-reducing early salpingectomy and delayed oophorectomy. While the initial results are expected in the near future, the long-term HRUS outcomes will take several years [52]. Other prospective studies are due to report on long-term QoL outcomes after RRSO and risk-reducing early salpingectomy [62]. Prospective trials of RRM are awaited. Until prospective studies using the EQ-5D are reported, the present study likely represents the only available HRUSs in the literature for RRM and RRSO sourced from patients who have undergone the procedures.

## 5. Conclusions

This mapping study finds that RRM is associated with a long-term disutility of −0.08 and an adjusted utility score of 0.92, whilst RRSO is associated with a long-term disutility of −0.03 and an adjusted utility score of 0.97. These values are highly relevant to patients at high risk of breast and ovarian cancer and for health-economic evaluations of cancer prevention. There is a need for high-quality prospective studies using the EQ-5D in these cohorts. 

## Figures and Tables

**Table 1:** RRM and RRSO cohorts with unadjusted HRUSs for surgical and control arms.

				Risk-Reducing Surgical Arm				Control Arm/Population Reference					
Study	Country	Sample Description	Source Data	*n*	Age (years)	HRUS	95% CI	Source	*n*	Age (years)	HRUS	95% CI	SE
Bai 2019 [41]	Sweden	RRM < 2 yr ^#^	SF-36	92	42.2	0.89	0.85–0.93	Teni 2022 [38]		40–44	0.92	0.74–1.00	
		RRM > 2 yr ^#^		92	52.7	0.85	0.81–0.90	Teni 2022 [38]		50–54	0.90	0.68–1.00	
Spindler 2021 [42]	Germany	RRM > 2 yr	SF-36	22	40.1	0.82	0.73–0.90	Szende 2014 [39]		35–44	0.96		0.005
Gopie 2013 [43]	Netherlands	RRM < 2 yr	SF-36	48	37.1	0.86	0.80–0.92	Szende 2014 [39]		35–44	0.92		0.014
Miseré 2022 [44]	Netherlands	RRM > 2 yr auto.	SF-36	33	42.6	0.85	0.78–0.93	Szende 2014 [39]		35–44	0.92		0.014
		RRM > 2 yr impl.		14	33	0.81	0.70–0.91	Szende 2014 [39]		25–34	0.91		0.011
Chae 2021 [45]	South Korea	RRSO > 1 yr ^#^	SF-36	30	49.8	0.83	0.74–0.91	Control arm	22	42.1	0.93	0.86–1.00	
		RRSO > 1 yr pre. ^#^		16	NR	0.89	0.79–0.98	Control arm	22	42.1	0.93	0.86–1.00	
		RRSO > 1 yr post. ^#^		14	NR	0.76	0.62–0.90	Control arm	22	42.1	0.93	0.86–1.00	
Finch 2013 [51]	Canada	RRSO > 1 yr pre.	SF-12	63	44.7	0.82	0.76–0.88	Yan 2023 [40]		35–44	0.88	0.68–1.00	
		RRSO > 1 yr post.		30	52.7	0.77	0.69–0.85	Yan 2023 [40]		45–54	0.86	0.62–1.00	
Fang 2009 [46]	US	RRSO < 1 yr ^#^	SF-36	38	46.0	0.85	0.78–0.92	Control arm	37	46.0	0.86	0.78–0.93	
		RRSO > 1 yr ^#^		38	46.0	0.83	0.75–0.90	Control arm	37		0.86	0.78–0.93	
Mai 2020 [47]	US	RRSO > 1 yr ^#^	SF-36	313	48.6	0.84	0.82–0.87	Control arm	586	47.6	0.85	0.83–0.87	
		RRSO < 1 yr ^#^		528	48.6	0.82	0.80–0.84	Control arm	952	47.6	0.86	0.84–0.87	
Robson 2003 [48]	US	RRSO >1 yr	SF-36	53	51.2	0.83	0.77–0.89	Szende 2014 [39]		45–54	0.85		0.003

HRUSs for surgical arms are obtained by mapping; HRUSs for control arms are obtained by mapping or from population references. Cohorts are presented by subgroup. Auto—autologous reconstruction. CI—confidence interval; HRUSs—health-related utility scores; impl.—implant reconstruction; n—number of participants; RRM—risk-reducing mastectomy; SE—standard error; pre.—pre-menopausal; post.—post-menopausal; yr—year of follow-up; ^#^ indicates same patient cohort across short-term and long-term follow-up.

**Table 2:** Unadjusted and adjusted disutilities and utility scores for RRM and RRSO by subgroup.

		Unadjusted Utility Scores				Adjusted Disutility (Additive Approach)				Adjusted Utility Scores (Multiplicative Approach)		
Subgroup	No. of Studies	*n*	I^2^ (%)	HRUS	95% CI	*n*	I^2^ (%)	Disutility	95% CI	I^2^ (%)	HRUS	95% CI
RRM >2 yrs	3	161	0.0	0.84	0.81–0.87	322	31.4	−0.08	−0.11–−0.04	33.2	0.92	0.88–0.95
RRM < 2yrs	2	140	0.0	0.88	0.85–0.91	280	0.0	−0.04	−0.07–0.00	0.0	0.96	0.92–1.00
RRSO > 1yr	5	527	0.0	0.83	0.81–0.85	1318	17.2	−0.03	−0.05–0.00	34.0	0.97	0.94–0.99
RRSO < 1yr	2	566	0.0	0.82	0.80–0.84	1555	0.0	−0.04	−0.06–−0.01	0.0	0.96	0.93–0.98
RRSO pre.	2	79	30.8	0.84	0.79–0.89	164	0.0	−0.05	−0.11–0.00	0.0	0.94	0.89–0.99
RRSO post.	2	44	0.0	0.77	0.70–0.83	96	0.0	−0.11	−0.19–−0.04	0.0	0.87	0.90–0.95

CI—confidence interval; HRUS—health-related utility score; n—number of participants (note that participant numbers are the same in both the additive and multiplicative approaches); RRM—risk-reducing mastectomy; RRSO—risk-reducing salpingo-oophorectomy; pre.—pre-menopausal; post.—post-menopausal; yr—year of follow-up.

## Data Availability

The original contributions presented in this study are included in the article/supplementary material; further inquiries can be directed to the corresponding author.

## Supplementary Materials

The following supporting information can be downloaded at https://www.mdpi.com/article/10.3390/cancers16071358/s1: Figure S1. Forest plot of risk-reducing mastectomy (RRM) with over 2 years’ follow-up, adjusted disutility (additive approach) (a), adjusted utility scores (multiplicative approach) (b) and risk-reducing salpingo-oophorectomy (RRSO) with over 1 year follow-up, adjusted disutility (additive approach) (c) and adjusted utility scores (multiplicative approach) (d).

## References

[1] Manchanda R., Sideris M. (2022). Population-based genetic testing for cancer susceptibility genes: Quo vadis?. BJOG.

[2] Venkatesan P. (2024). BRCA testing launched for people of Jewish ancestry in England. Lancet Oncol..

[3] Greenberg R., Aharonov-Majar E., Isakov O., Hayek S., Elefant N., Balicer R.D., Berliner Senderey A., Ben-Shachar S. (2023). Carrier screening program for BRCA1/BRCA2 pathogenic variants among Ashkenazi Jewish women in Israel: An observational study. Genet. Med. Open.

[4] NICE (2024). Ovarian Cancer: Identifying and Managing Familial and Genetic Risk. NICE Guideline [NG241]. https://www.nice.org.uk/guidance/ng241.

[5] Manchanda R., Blyuss O., Gaba F., Gordeev V.S., Jacobs C., Burnell M., Gan C., Taylor R., Turnbull C., Legood R. (2018). Current detection rates and time-to-detection of all identifiable BRCA carriers in the Greater London population. J. Med. Genet..

[6] NICE Familial Breast Cancer: Classification, Care and Managing Breast Cancer and Related Risks in People with a Family History of Breast Cancer Clinical Guideline [CG164]. https://www.nice.org.uk/guidance/cg164/chapter/Recommendations#risk-reduction-and-treatment-strategies.

[7] Sessa C., Balmaña J., Bober S., Cardoso M.-J., Colombo N., Curigliano G., Domchek S., Evans D., Fischerova D., Harbeck N. (2023). Risk reduction and screening of cancer in hereditary breast-ovarian cancer syndromes: ESMO Clinical Practice Guideline☆. Ann. Oncol..

[8] Li X., You R., Wang X., Liu C., Xu Z., Zhou J., Yu B., Xu T., Cai H., Zou Q. (2016). Effectiveness of Prophylactic Surgeries in BRCA1 or BRCA2 Mutation Carriers: A Meta-analysis and Systematic Review. Clin. Cancer Res..

[9] Rebbeck T.R., Friebel T., Lynch H.T., Neuhausen S.L., van ‘t Veer L., Garber J.E., Evans G.R., Narod S.A., Isaacs C., Matloff E. (2004). Bilateral prophylactic mastectomy reduces breast cancer risk in BRCA1 and BRCA2 mutation carriers: The PROSE Study Group. J. Clin. Oncol..

[10] Ludwig K.K., Neuner J., Butler A., Geurts J.L., Kong A.L. (2016). Risk reduction and survival benefit of prophylactic surgery in BRCA mutation carriers, a systematic review. Am. J. Surg..

[11] Crosbie E.J., Flaum N., Harkness E.F., Clayton R.D., Holland C., Martin-Hirsch P., Wood N., Keating P., Woodward E.R., Lalloo F. (2021). Specialist oncological surgery for removal of the ovaries and fallopian tubes in BRCA1 and BRCA2 pathogenic variant carriers may reduce primary peritoneal cancer risk to very low levels. Int. J. Cancer.

[12] Gaba F., Blyuss O., Tan A., Munblit D., Oxley S., Khan K., Legood R., Manchanda R. (2023). Breast Cancer Risk and Breast-Cancer-Specific Mortality following Risk-Reducing Salpingo-Oophorectomy in BRCA Carriers: A Systematic Review and Meta-Analysis. Cancers.

[13] Manchanda R., Gaba F., Talaulikar V., Pundir J., Gessler S., Davies M., Menon U., on behalf of the Royal College of Obstetricians and Gynaecologistsiley (2021). Risk-Reducing Salpingo-Oophorectomy and the Use of Hormone Replacement Therapy Below the Age of Natural Menopause: Scientific Impact Paper No. 66. BJOG.

[14] Gaba F., Manchanda R. (2020). Systematic review of acceptability, cardiovascular, neurological, bone health and HRT outcomes following risk reducing surgery in BRCA carriers. Best Pract. Res. Clin. Obstet. Gynaecol..

[15] NICE (2022). NICE Health Technology Evaluations: The Manual. https://www.nice.org.uk/process/pmg36/chapter/introduction-to-health-technology-evaluation.

[16] Simões Corrêa Galendi J., Vennedey V., Kentenich H., Stock S., Müller D. (2021). Data on Utility in Cost–Utility Analyses of Genetic Screen-and-Treat Strategies for Breast and Ovarian Cancer. Cancers.

[17] Wei X., Oxley S., Sideris M., Kalra A., Sun L., Yang L., Legood R., Manchanda R. (2022). Cost-Effectiveness of Risk-Reducing Surgery for Breast and Ovarian Cancer Prevention: A Systematic Review. Cancers.

[18] Manchanda R., Legood R., Pearce L., Menon U. (2015). Defining the risk threshold for risk reducing salpingo-oophorectomy for ovarian cancer prevention in low risk postmenopausal women. Gynecol. Oncol..

[19] Manchanda R., Legood R., Antoniou A.C., Gordeev V.S., Menon U. (2016). Specifying the ovarian cancer risk threshold of ‘premenopausal risk-reducing salpingo-oophorectomy’ for ovarian cancer prevention: A cost-effectiveness analysis. J. Med. Genet..

[20] Sanders G.D., Neumann P.J., Basu A., Brock D.W., Feeny D., Krahn M., Kuntz K.M., Meltzer D.O., Owens D.K., Prosser L.A. (2016). Recommendations for conduct, methodological practices, and reporting of cost-effectiveness analyses: Second panel on cost-effectiveness in health and medicine. Jama.

[21] Grann V.R., Jacobson J.S., Sundararajan V., Albert S.M., Troxel A.B., Neugut A.I. (1999). The quality of life associated with prophylactic treatments for women with BRCA1/2 mutations. Cancer J. Sci. Am..

[22] Grann V.R., Patel P., Bharthuar A., Jacobson J.S., Warner E., Anderson K., Tsai W.Y., Hill K.A., Neugut A.I., Hershman D. (2010). Breast cancer-related preferences among women with and without BRCA mutations. Breast Cancer Res. Treat..

[23] Matza L.S., Stewart K.D., Lloyd A.J., Rowen D., Brazier J.E. (2021). Vignette-Based Utilities: Usefulness, Limitations, and Methodological Recommendations. Value Health.

[24] Rowen D., Brazier J., Wong R., Wailoo A. (2020). Measuring and valuing health-related quality of life when sufficient EQ-5D data is not available. NICE DSU Rep..

[25] Wei X., Oxley S., Sideris M., Kalra A., Brentnall A., Sun L., Yang L., Legood R., Manchanda R. (2023). Quality of life after risk-reducing surgery for breast and ovarian cancer prevention: A systematic review and meta-analysis. Am. J. Obstet. Gynecol..

[26] Ware J.E. (2000). SF-36 health survey update. Spine.

[27] Ware J., Kosinski M., Keller S. (1998). SF-12: How to Score the SF-12 Physical and Mental Health Summary Scales.

[28] Gandek B., Ware J.E., Aaronson N.K., Apolone G., Bjorner J.B., Brazier J.E., Bullinger M., Kaasa S., Leplege A., Prieto L. (1998). Cross-validation of item selection and scoring for the SF-12 Health Survey in nine countries: Results from the IQOLA Project. International Quality of Life Assessment. J. Clin. Epidemiol..

[29] Dakin H., Abel L., Burns R., Yang Y. (2018). Review and critical appraisal of studies mapping from quality of life or clinical measures to EQ-5D: An online database and application of the MAPS statement. Health Qual. Life Outcomes.

[30] Ware J., Ma K., Keller S.D. (1993). SF-36 Physical and Mental Health Summary Scales: A User’s Manual.

[31] Taft C., Karlsson J., Sullivan M. (2001). Do SF-36 summary component scores accurately summarize subscale scores?. Qual. Life Res..

[32] Brazier J.E., Yang Y., Tsuchiya A., Rowen D.L. (2010). A review of studies mapping (or cross walking) non-preference based measures of health to generic preference-based measures. Eur. J. Health Econ..

[33] Lawrence W.F., Fleishman J.A. (2004). Predicting EuroQoL EQ-5D preference scores from the SF-12 Health Survey in a nationally representative sample. Med. Decis. Mak..

[34] Rowen D., Brazier J., Roberts J. (2009). Mapping SF-36 onto the EQ-5D index: How reliable is the relationship?. Health Qual. Life Outcomes.

[35] Crott R. (2014). Mapping algorithms from QLQ-C30 to EQ-5D utilities: No firm ground to stand on yet. Expert Rev. Pharmacoeconomics Outcomes Res..

[36] Brazier J., Connell J., Papaioannou D., Mukuria C., Mulhern B., Peasgood T., Jones M.L., Paisley S., O’Cathain A., Barkham M. (2014). A systematic review, psychometric analysis and qualitative assessment of generic preference-based measures of health in mental health populations and the estimation of mapping functions from widely used specific measures. Health Technol. Assess..

[37] Khan I., Morris S., Pashayan N., Matata B., Bashir Z., Maguirre J. (2016). Comparing the mapping between EQ-5D-5L, EQ-5D-3L and the EORTC-QLQ-C30 in non-small cell lung cancer patients. Health Qual. Life Outcomes.

[38] Teni F.S., Gerdtham U.-G., Leidl R., Henriksson M., Åström M., Sun S., Burström K. (2022). Inequality and heterogeneity in health-related quality of life: Findings based on a large sample of cross-sectional EQ-5D-5L data from the Swedish general population. Qual. Life Res..

[39] Janssen B., Szende A. (2014). Population Norms for the EQ-5D.

[40] Yan J., Xie S., Johnson J.A., Pullenayegum E., Ohinmaa A., Bryan S., Xie F. (2023). Canada population norms for the EQ-5D-5L. Eur. J. Health Econ..

[41] Bai L., Arver B., Johansson H., Sandelin K., Wickman M., Brandberg Y. (2019). Body image problems in women with and without breast cancer 6-20 years after bilateral risk-reducing surgery—A prospective follow-up study. Breast.

[42] Spindler N., Ebel F., Briest S., Wallochny S., Langer S. (2021). Quality of life after bilateral risk-reducing mastectomy and simultaneous reconstruction using pre-pectoral silicone implants. Patient Prefer. Adherence.

[43] Gopie J.P., Mureau M.A., Seynaeve C., Ter Kuile M.M., Menke-Pluymers M.B., Timman R., Tibben A. (2013). Body image issues after bilateral prophylactic mastectomy with breast reconstruction in healthy women at risk for hereditary breast cancer. Fam. Cancer.

[44] Miseré R.M., Joosen M.E., Claassens E.L., de Grzymala A.A.P., Heuts E.M., van der Hulst R.R. (2022). Patient-reported outcomes following bilateral prophylactic mastectomy and immediate breast reconstruction: Comparing implant-based with autologous breast reconstruction. Eur. J. Plast. Surg..

[45] Chae S., Kim E.K., Jang Y.R., Lee A.S., Kim S.K., Suh D.H., Kim K., No J.H., Kim Y.B., Kim S.W. (2021). Effect of risk-reducing salpingo-oophorectomy on the quality of life in Korean BRCA mutation carriers. Asian J. Surg..

[46] Fang C.Y., Cherry C., Devarajan K., Li T., Malick J., Daly M.B. (2009). A prospective study of quality of life among women undergoing risk-reducing salpingo-oophorectomy versus gynecologic screening for ovarian cancer. Gynecol. Oncol..

[47] Mai P.L., Huang H.Q., Wenzel L.B., Han P.K., Moser R.P., Rodriguez G.C., Boggess J., Rutherford T.J., Cohn D.E., Kauff N.D. (2020). Prospective follow-up of quality of life for participants undergoing risk-reducing salpingo-oophorectomy or ovarian cancer screening in GOG-0199: An NRG Oncology/GOG study. Gynecol. Oncol..

[48] Robson M., Hensley M., Barakat R., Brown C., Chi D., Poynor E., Offit K. (2003). Quality of life in women at risk for ovarian cancer who have undergone risk-reducing oophorectomy. Gynecol. Oncol..

[49] Nebgen D.R., Hurteau J., Holman L.L., Bradford A., Munsell M.F., Soletsky B.R., Sun C.C., Chisholm G.B., Lu K.H. (2018). Bilateral salpingectomy with delayed oophorectomy for ovarian cancer risk reduction: A pilot study in women with BRCA1/2 mutations. Gynecol. Oncol..

[50] Finch A., Metcalfe K.A., Chiang J.K., Elit L., McLaughlin J., Springate C., Demsky R., Murphy J., Rosen B., Narod S.A. (2011). The impact of prophylactic salpingo-oophorectomy on menopausal symptoms and sexual function in women who carry a BRCA mutation. Gynecol. Oncol..

[51] Finch A., Metcalfe K.A., Chiang J., Elit L., McLaughlin J., Springate C., Esplen M.J., Demsky R., Murphy J., Rosen B. (2013). The impact of prophylactic salpingo-oophorectomy on quality of life and psychological distress in women with a BRCA mutation. Psycho-Oncol..

[52] Gaba F., Robbani S., Singh N., McCluggage W.G., Wilkinson N., Ganesan R., Bryson G., Rowlands G., Tyson C., Arora R. (2021). Preventing Ovarian Cancer through early Excision of Tubes and late Ovarian Removal (PROTECTOR): Protocol for a prospective non-randomised multi-center trial. Int. J. Gynecol. Cancer.

[53] Gaba F., Goyal S., Marks D., Chandrasekaran D., Evans O., Robbani S., Tyson C., Legood R., Saridogan E., McCluggage W.G. (2021). Surgical decision making in premenopausal BRCA carriers considering risk-reducing early salpingectomy or salpingo-oophorectomy: A qualitative study. J. Med. Genet..

[54] Steenbeek M.P., Harmsen M.G., Hoogerbrugge N., de Jong M.A., Maas A., Prins J.B., Bulten J., Teerenstra S., van Bommel M.H.D., van Doorn H.C. (2021). Association of Salpingectomy With Delayed Oophorectomy Versus Salpingo-oophorectomy With Quality of Life in BRCA1/2 Pathogenic Variant Carriers: A Nonrandomized Controlled Trial. JAMA Oncol..

[55] Hickey I., Jha S., Wyld L. (2020). The psychosexual effects of risk reducing bilateral salpingooophorectomy in female BRCA1/2 mutation carriers: A systematic review. Eur. J. Cancer.

[56] Gaba F., Blyuss O., Chandrasekaran D., Osman M., Goyal S., Gan C., Izatt L., Tripathi V., Esteban I., McNicol L. (2021). Attitudes towards risk-reducing early salpingectomy with delayed oophorectomy for ovarian cancer prevention: A cohort study. BJOG.

[57] Hunt K.K., Euhus D.M., Boughey J.C., Chagpar A.B., Feldman S.M., Hansen N.M., Kulkarni S.A., McCready D.R., Mamounas E.P., Wilke L.G. (2017). Society of surgical oncology breast disease working group statement on prophylactic (risk-reducing) mastectomy. Ann. Surg. Oncol..

[58] Metcalfe K., Foulkes W., Kim-Sing C., Ainsworth P., Rosen B., Armel S., Poll A., Eisen A., Gilchrist D., Chudley A. (2008). Family history as a predictor of uptake of cancer preventive procedures by women with a BRCA1 or BRCA2 mutation. Clin. Genet..

[59] Padamsee T.J., Wills C.E., Yee L.D., Paskett E.D. (2017). Decision making for breast cancer prevention among women at elevated risk. Breast Cancer Res..

[60] Hanson H., Kulkarni A., Loong L., Kavanaugh G., Torr B., Allen S., Ahmed M., Antoniou A.C., Cleaver R., Dabir T. (2022). UK consensus recommendations for clinical management of cancer risk for women with germline pathogenic variants in cancer predisposition genes: RAD51C, RAD51D, BRIP1 and PALB2. J. Med. Genet..

[61] Wei X., Sun L., Slade E., Fierheller C.T., Oxley S., Kalra A., Sia J., Sideris M., McCluggage W.G., Bromham N. (2024). Cost-Effectiveness of Gene-Specific Prevention Strategies for Ovarian and Breast Cancer. JAMA Netw. Open.

[62] Harmsen M.G., Arts-de Jong M., Hoogerbrugge N., Maas A.H., Prins J.B., Bulten J., Teerenstra S., Adang E.M., Piek J.M., van Doorn H.C. (2015). Early salpingectomy (TUbectomy) with delayed oophorectomy to improve quality of life as alternative for risk-reducing salpingo-oophorectomy in BRCA1/2 mutation carriers (TUBA study): A prospective non-randomised multicentre study. BMC Cancer.

