# Supplementary material for: Utility Scores for Risk-Reducing Mastectomy and Risk-Reducing Salpingo-Oophorectomy: Mapping to EQ-5D
Source: Cancers (Basel). 2024 Mar 30;16(7):1358. doi: 10.3390/cancers16071358 (PMC11010846; doi:10.3390/cancers16071358)
Supplement: Supplementary file 1 [file cancers-16-01358-s001.zip › cancers-2928125-supplementary.pdf]

Supplementary File S1

Figure S1a: RRM over 2 years' follow-up, adjusted disutility (additive approach)

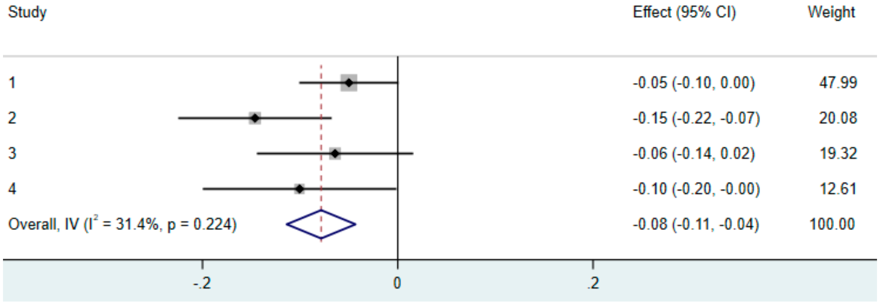

Figure S1b: RRM over 2 years' follow-up, adjusted utility score (multiplicative approach)

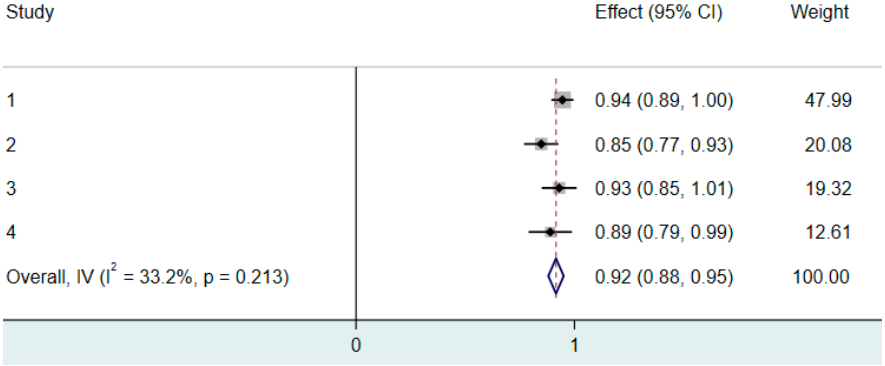

Figure S1c: RRSO over 1 year follow-up, adjusted disutility (additive approach)

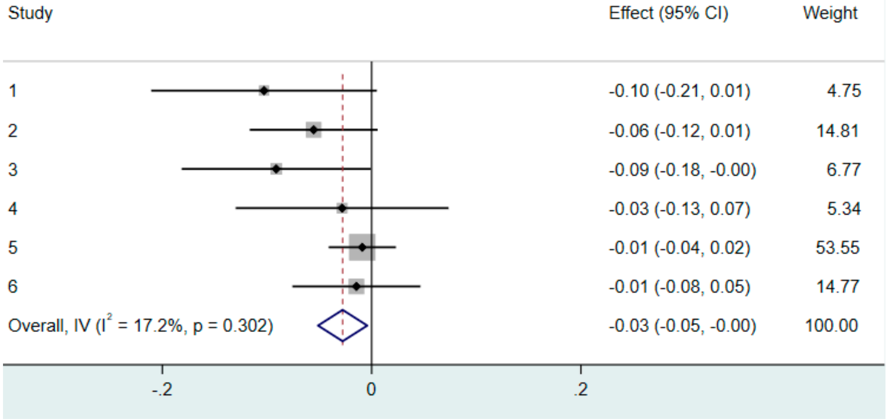

Figure S1d: RRSO over 1 year follow-up, adjusted utility score (multiplicative approach)

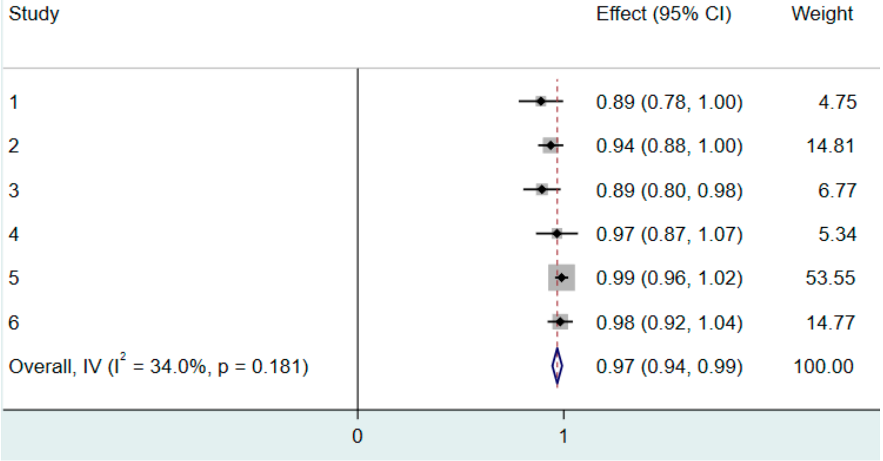

Forest plot of risk-reducing mastectomy (RRM) with over 2 years' follow-up, adjusted disutility (additive approach) (S1a), adjusted utility scores (multiplicative approach) (S1b) and risk-reducing salpingo-oophorectomy (RRSO) with over 1 year follow-up, adjusted disutility (additive approach) (S1c) and adjusted utility scores (multiplicative approach) (S1d).
